# Analysis of risk factors associated with the high incidence of amblyopia in preterm infants at the corrected gestational age of 12 months

**DOI:** 10.1186/s12887-023-03937-y

**Published:** 2023-03-25

**Authors:** Yiwen Cao, Ying Wang, Bian Li, Dehai Zhu, Tian Sang, Xueyan Du, Wanjun Shi, Liu Yang

**Affiliations:** 1 Department of Pediatric Ophthalmology, Peking University First Hospital, Beijing, 100034 China; 2 Department of Pediatric, Peking University First Hospital, Beijing, 100034 China; 3 Department of Ophthalmology, Peking University First Hospital, Beijing, 100034 China

**Keywords:** Amblyopia risk factors, Preterm infants, Bronchopulmonary dysplasia, White matter injury, Levothyroxine, Invasive ventilator, Glucocorticoids, Fine motor

## Abstract

**Objective:**

To investigate the perinatal and in-hospital risk factors associated with the high incidence of amblyopia in preterm infants and to analyze the correlation between the amblyopia and neurodevelopment.

**Methods:**

Children discharged from the neonatal intensive care unit (NICU) at 12 months of corrected gestational age were retrospectively included in this study. Ocular screening was performed in children. At the risk of amblyopia was determined according to the American Academy of Ophthalmology Guidelines for automated preschool vision screening factors. Differences in perinatal characteristics, complications during hospitalization, and treatment modalities between the two groups of children were analyzed, and multifactorial logistic regression analysis was used to identify the independent risk factors for amblyopia. The results of developmental assessment were collected retrospectively to analyze the correlation between amblyopia and various aspects of neurological development.

**Results:**

A total of 128 preterm infants, 30 in the amblyopia risk group and 98 in the non-amblyopia risk group, were included in this study. Univariate analysis showed that the amblyopia risk group had lower birth weights, higher rates of asphyxia, preterm brain white matter injury, bronchopulmonary dysplasia (BPD), intraventricular hemorrhage (IVH), sepsis during hospitalization, and higher rates of treatment with pulmonary surfactant (PS), blood transfusion, invasive ventilator, and levothyroxine. Logistic regression analysis showed that BPD in the neonatal period (odds ratio [OR] 8.355, 95% confidence interval [CI] 1.492, 46.786), brain white matter injury (OR 16.742, 95% CI 0.684, 409.804), treatment with levothyroxine (OR 2.859, 95% CI 0.946, 8.639), and use of an invasive ventilator (OR 2.983, 95% CI 0.942, 9.445) were independent risk factors for amblyopia at 12 months of corrected gestational age, while the administration of glucocorticoids (OR 0.055, 95% CI 0.004, 0.737) was a protective factor. Regarding neurodevelopmental assessment, the number of infants with lagging fine motor development was greater in the amblyopia risk group.

**Conclusion:**

The presence of BPD in the neonatal period, brain white matter damage in preterm infants, and use of levothyroxine and invasive ventilator were high risk factors for amblyopia. The use of glucocorticoids therapy was a protective factor. Children with risk of amblyopia had a higher rate of poor fine motor development.

## Background

Amblyopia is one of the leading causes of visual impairment in preschool children in China [1, 2]. The prevalence of amblyopia is 1.44% globally and 1.09% in Asia [3]. Late diagnosis and failure to provide targeted treatment of amblyopia can lead to lifelong vision loss. However, amblyopia is a reversible condition and clinical studies have shown that early detection and regular treatment of amblyopia can significantly improve the vision [4] and improve the quality of life of the affected children. The earlier the age of commencing amblyopia treatment, the better the response to it [5].

With medical advances and improvements in obstetrics and neonatal intensive care, the survival rate of preterm births continues to increase and preterm births now account for 12% of live births worldwide; however, prematurity is also associated with one of the highest rates of disability [6], including visual dysfunction. Prematurity and low birth weight have been reported in the literature as risk factors for amblyopia [7, 8]. Yassin et al. showed that the prevalence of amblyopia in preterm infants was as high as 10% [9]. Therefore, clinicians should be able to analyze the clinical characteristics of preterm infants in the neonatal period to identify the risk factors for amblyopia, thus ensuring active follow-up and timely treatment of the at-risk infants.

Instrument-based photoscreening is a quick method to evaluate a child’s eye condition and is widely used for vision screening in children [10, 11]. In the 2017 Pediatric Eye Evaluations Preferred Practice Pattern (PPP) [12] guidelines, it is recommended that children undergo instrument-based photoscreening annually, starting at age 1 year, until the child is ready for vision screening. “Spot” is a hand-held infrared photoscreener (Welch Allyn, Skaneateles Falls, NY) that has been used extensively in recent years. Examination with Spot does not require dilated pupils and requires the child to look at the instrument at a distance of 1 m for approximately 2 s to identify important amblyopic risk factors in the child’s eye, including refractive parameters and eye position. The distance of the instrument from the child keeps the examination time short and the child’s cooperation high, making it particularly suitable for ocular examination during the post-discharge follow-up of preterm infants.

In this study, the clinical characteristics of preterm infants hospitalized in a single-center neonatal intensive care unit (NICU) ward of a tertiary hospital in Beijing were summarized in the neonatal period, and regular longitudinal follow-up was performed until 12 months of corrected gestational age to detect amblyopia risk factors (ARFs) and to assess neurodevelopmental scales. The purpose of this study was to investigate the risk factors associated with amblyopia during hospitalization and in the neonatal period in preterm infants at 12 months of corrected gestational age, and their relationship with neurodevelopmental prognosis to guide the clinical follow-up of preterm infants in the NICU, to detect ARFs in a timely manner, to further examine and treat children at risk, to reduce the occurrence of amblyopia, to reduce vision-related neurological disability, and to provide clinical evidence for the relationship between the presence of ARFs and neurodevelopment in preterm infants at the corrected gestational age of 12 months.

## Subjects and methods

### Subjects

This was a retrospective study of preterm infants admitted to the NICU at Peking University First Hospital from July 2018 to September 2020, who were discharged in a stable condition. Regular follow-up was performed after discharge at the corrected gestational ages of 40 weeks and 1, 3, 6, 9, and 12 months. Ocular Spot photoscreening and Gesell Neurodevelopmental Scale assessment were performed at 12 months of corrected gestational age. The study data were obtained from the children’s medical records during hospitalization and outpatient follow-up. This study was approved by the Ethics Committee of Peking University First Hospital (2021Yan281). The data collection process was in accordance with the Helsinki Declaration.

The inclusion criteria were as follows: (1) gestational age at birth between 25^+ 1^ weeks to 36^+ 6^ weeks and (2) admission to the NICU of Peking University First Hospital from July 2018 to September 2020.

The exclusion criteria were as follows: (1) survival < 12 months of the corrected gestational age for various reasons; (2) social factors for forgoing treatment; (3) failure to screen for the amblyopia risk using the Spot photoscreener during follow-up till 12 months of corrected gestational age; (4) failure to evaluate using the Gesell Neurodevelopmental Scale at 12 months of corrected positive gestational age; and (5) incomplete available information pertaining to the perinatal period and hospitalization of preterm infants for various reasons.

### Amblyopia risk determination

**Fig. 1:**
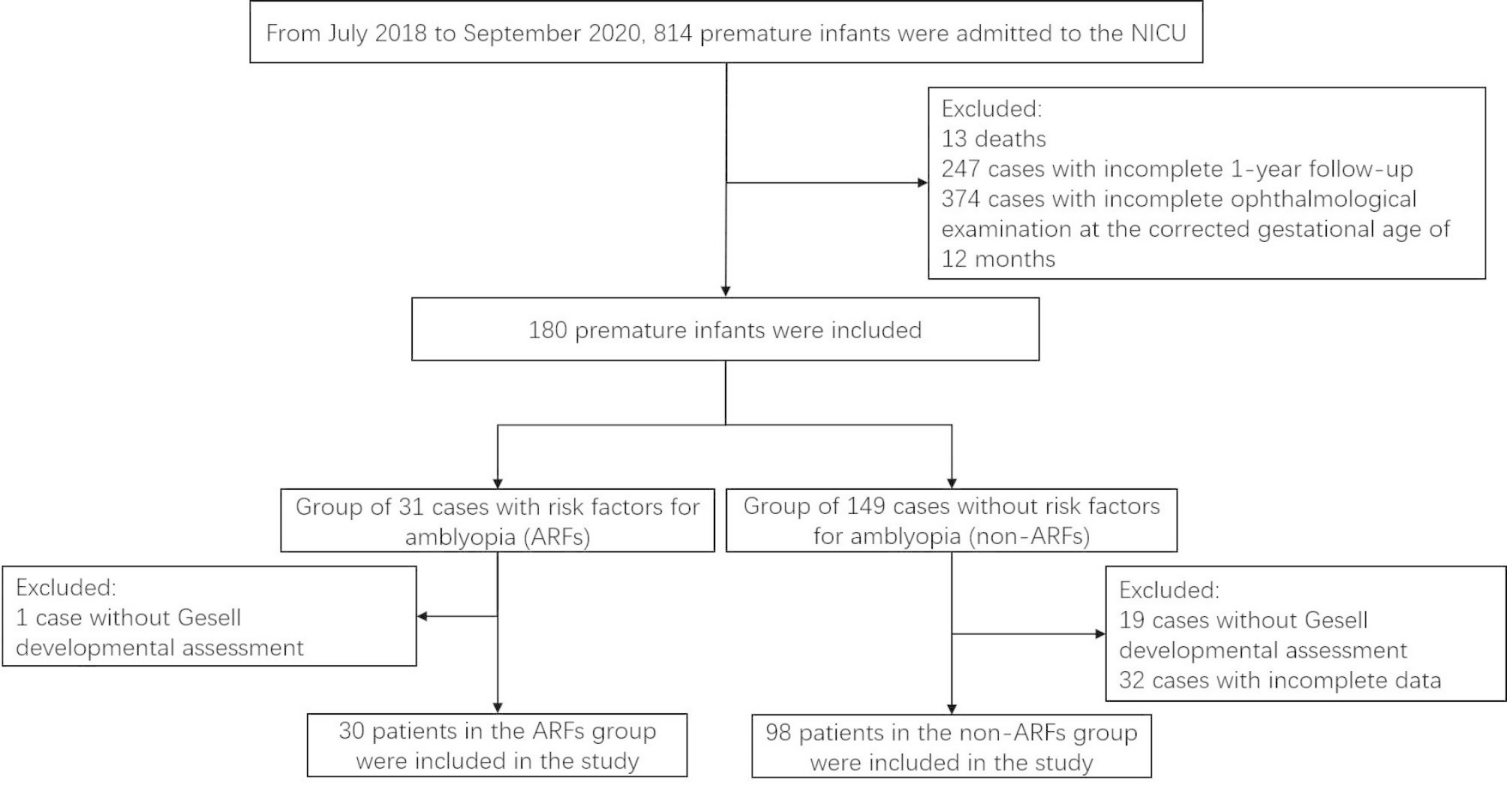
Flow chart for inclusion of the cases. ARFs, amblyopia risk factors. NICU, neonatal intensive care unit

Amblyopia risk factors for all children were determined based on the screening results of the Spot photoscreener (Welch Allyn, Skaneateles Falls, NY) at 1 year of age. Screening was preformed by trained professionals. The specific criteria were based on the American Academy of Ophthalmology Guidelines for automated preschool vision screening [13], which were determined as astigmatism > 2.00 D, hyperopia > 4.50 D, myopia > 3.00 D, anisometropia > 2.50 D, and the presence of manifest strabismus > 8 PD in the primary position in children 12–30 months of age. Instrumental screening defined the children as at risk for amblyopia if they met the criteria and control children if they did not. The flow chart is shown in Fig. 1.

### Analyzed data

1. Demographic data: sex, gestational age at birth, birth weight, age of the parents, small for gestational age (SGA), and large for gestational age (LGA).
2. Clinical characteristics of the preterm infants.
  1. Maternal pregnancy comorbidities: gestational hypertensive syndrome and gestational diabetes mellitus (GDM); and singleton or multiple births.
  2. Conditions associated with hospitalization: (1) neonatal respiratory distress syndrome (NRDS); (2) bronchopulmonary dysplasia (BPD); (3) intraventricular hemorrhage (IVH) and posthemorrhagic hydrocephalus; (4) electroencephalogram(EEG) abnormalities; (5) sepsis; (6) bacterial meningitis; (7) necrotizing enterocolitis (NEC); and (8) retinopathy of prematurity (ROP): examination with 0.5% compound tropicamide for pupil dilatation was performed by an experienced clinician using an indirect inspection lens to check for the presence of ROP.
  3. Treatment during hospitalization: (1) use of more than one pulmonary surfactant (PS) replacement therapy; (2) continued treatment with hormones for BPD for at least one full course; (3) treatment with a ventilator during hospitalization, including an invasive ventilator or noninvasive ventilator; (4) blood transfusion; (5) surgical treatment, such as bowel surgery for NEC during hospitalization; and (6) supplemental treatment with levothyroxine for hypothyroidism.
  4. Total length of stay in the neonatal care unit for premature infants.

3. Assessment of neurological development.

All children were assessed at 1 year of age using the Gesell Developmental Inventory. Assessments were conducted by 2–3 trained professionals from the same assessment team and there was consistency in scoring results after prestudy evaluation. The results were analyzed in five areas: adaptive, gross motor, fine motor, language, and personal socialization, and the outcome scores were judged as normal or abnormal. Normal was defined as a score of ≥ 85 for each ability area; abnormal was defined as a score of < 85.

### Statistical analyses

SPSS 15.0 statistical software (SPSS, IBM, Chicago, IL, USA). was used to analyze the data. The *χ2* test was used for comparison between groups and comparison of rates for single-factor analysis. Logistic regression was used for multi-factor analysis using the stepwise backward method, and the rejection criterion was 0.1. P < 0.05 was considered a statistically significant difference.

## Results

### General information

**Fig. 2:**
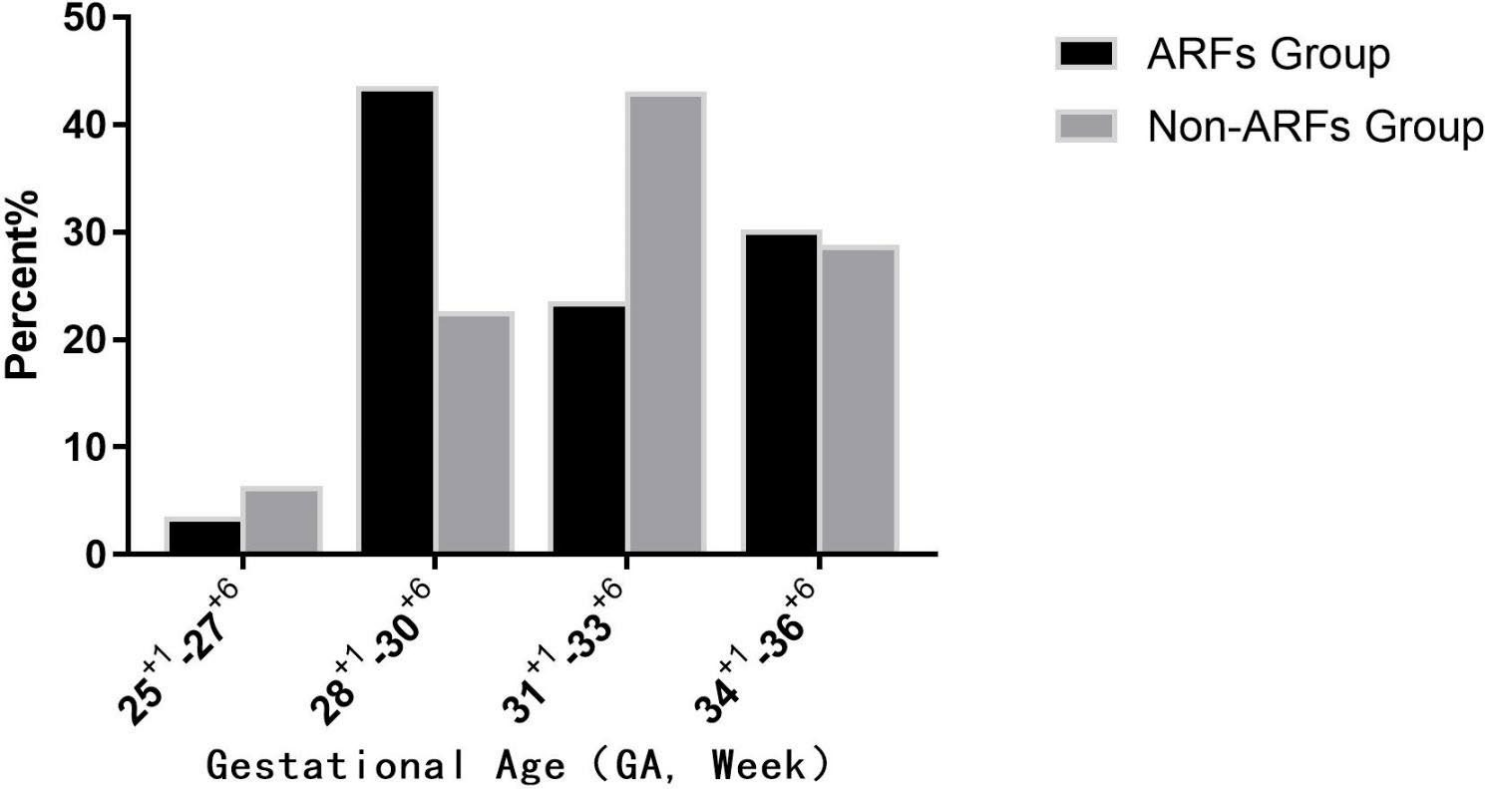
Distribution of gestational age among the included preterm infants. ARFs, amblyopia risk factors

A total of 128 preterm infants with a mean gestational age of 31.62 ± 2.523 weeks (25^+ 1^–36^+ 6^ weeks) were included in this study (Table 1; Fig. 1). Among the preterm infants, 78 were boys and 50 were girls; the mean birth weight was 1709.41 ± 543.718 g (660–3200 g) (Table 2; Fig. 2).

**Table 1:** Gestational age distribution among the included preterm infants

Gestational age (week)	ARF group (%)^a^	Non-ARF group (%)^a^	Total (%)^a^
25^+ 1^–27^+ 6^	1 (3.3)	6 (6.1)	7(5.5)
28^+ 1^–30^+ 6^	13 (43.3)	22 (22.4)	35 (27.3)
31^+ 1^–32^+ 6^	7 (23.3)	42 (42.9)	42 (32.8)
33^+ 1^–36^+ 6^	9 (30)	28 (28.6)	28 (21.9)
Total	30 (100)	98 (100)	128 (100)

Note: ^a^ expressed as a number (percentage)

ARFs, amblyopia risk factors.

**Table 2:** Distribution of birth weight among the included preterm infants

Birth weight	ARF group (%)^a^	Non-ARF group (%)^a^	Total (%)^a^
500–1000 g	6 (20)	5(5.1)	11 (8.6)
1001–1500 g	12 (40)	32 (32.7)	34 (34.4)
1501–2000 g	6 (20)	33 (33.7)	38 (30.5)
2001–2500 g	4 (13.3)	17 (17.3)	21 (16.4)
2500–3001 g	2 (6.7)	9 (9.2)	11 (8.6)
> 3000 g	0 (0)	2 (2.0)	2 (1.6)
Total	30 (100)	98 (100)	128 (100)

Note: ^a^ expressed as a number (percentage)

ARFs, amblyopia risk factors.

### Amblyopia risk assessment

The enrolled preterm infants were regularly followed up until 12 months of corrected gestational age. According to the Spot photoscreener, there were 30 participants in the amblyopia risk group (30/128, 23.4%), including 18 male individuals and 12 female individuals, and 98 participants in the non-amblyopia risk group (98/128, 76.6%), including 60 male individuals and 38 female individuals; there was no statistical difference in sex between the two groups (Table 3).

**Table 3:** Comparison of the general characteristics between the ARF and non-ARF groups

Demographic information		ARF group	Non-ARF group	Statistical value	P
Sex	M	18	60	0.014	0.904
	F	12	38		
Weeks of gestation (weeks)		31.40 ± 2.64	31.68 ± 2.49	0.537	0.592
Birth weight (g)		1533 ± 560.15	1763.41 ± 529.79	2.057	0.042

ARFs, amblyopia risk factors.

**Fig. 3:**
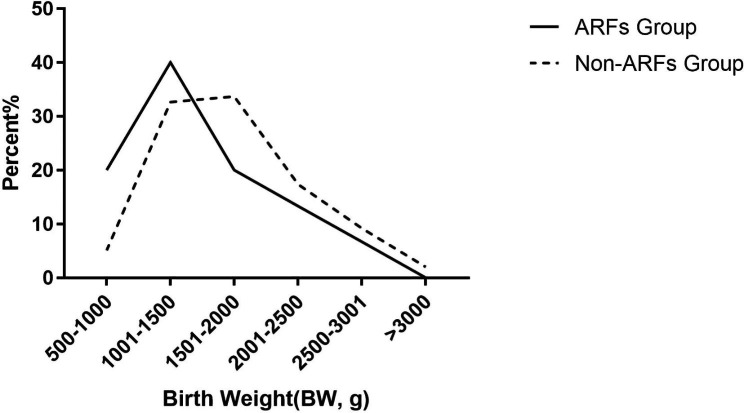
Birth weight distribution among the included preterm infants. ARFs, amblyopia risk factors

The mean gestational age was 31.10 ± 2.647 weeks in the amblyopia risk group and 31.68 ± 2.494 weeks in the non-amblyopia risk group, with no statistical difference in the gestational age between the two groups (Table 1; Fig. 2). The mean birth weight of 1533.00 ± 560.150 g in the amblyopia risk group was significantly lower than that of 1763.42 ± 529.794 g in the non-amblyopia risk group (P < 0.05) (Table 2; Fig. 3).

### Risk classification of amblyopia in preterm infants at the corrected gestational age of 12 months

Thirty of the 128 children were at risk for amblyopia (23.4%). Further classification of the amblyopia risk revealed 20 cases of astigmatism (15.63%), eight cases of strabismus (6.25%), seven cases of anisometropia (5.47%), four cases of hyperopia (3.13%), no cases of myopia (0.00%), and eight cases of mixed factors (6.25%). Based on the above data, astigmatism was found to be the major risk for amblyopia followed by strabismus (Table 4).

**Table 4:** Amblyopia risk classification in preterm infants at the corrected gestational age of 12 months

ARFs	N	Percentage (%)
Hyperopia	4	3.13
Astigmatism	20	15.63
Myopia	0	0.00
Anisometropia	7	5.47
Strabismus	8	6.25
Mixed factors	8	6.25
Totals	30	23.4

ARFs, amblyopia risk factors.

### Single factor analysis of risk factors associated with the risk of amblyopia in the neonatal period in preterm infants at the corrected gestational age of 12 months

#### General perinatal risk factors

There were no statistical differences in the age of the mother or father, presence of gestational hypertension or GDM during the pregnancy, whether the children were born as twins, and birth weight less than the gestational age (SGA) or greater than the gestational age (LGA) between the two groups of children (Table 5).

#### Risks associated with complications during NICU stay

Regarding complications during hospitalization, children in the ARFs group had higher rates of asphyxia, preterm brain white matter injury, BPD, IVH, and sepsis during neonatal hospitalization than the non-ARF group, and the differences were statistically significant. The incidence of NRDS, hydrocephalus, neonatal NEC, ROP meningitis, and EEG abnormalities was not statistically different among the hospitalized children (Table 5).

#### Risks associated with various treatments during NICU stay

During NICU stay of preterm infants, the rates of treatment with PS, blood transfusion, invasive ventilator use, and levothyroxine use were significantly higher in the ARF group than in the non-ARF group, with statistically significant differences. However, there were no statistically significant differences between the two groups in terms of whether glucocorticoids were used, sequential invasive plus noninvasive ventilator use, or whether surgery was performed during hospitalization (Table 5).

**Table 5:** One-way comparison of the risk factors in the amblyopia risk group versus the non-amblyopia risk group of preterm infants with corrected gestational age of 12 months

	Risk factor	ARF group	Non-ARF group	Statistical value	P
	Number of cases	30	98		
General perinatal factors	Age of mother (years)^a^	32.66 ± 3.49	33.26 ± 3.77	0.773	0.441
	Father’s age (years)^a^	33.86 ± 3.91	34.94 ± 4.68	1.148	0.253
	Maternal hyperemesis^b^	10 (33.3)	24 (24.5)	0.921	0.337
	GDM^b^	7 (23.3)	37 (37.8)	2.118	0.146
	Twin pregnancies^b^	8 (26.7)	30 (30.6)	0.171	0.679
	SGA^b^	5 (16.7)	6 (6.1)	3.251	0.071
	LGA^b^	0 (0)	2 (2.0)		1.000
Complications during hospitalization	Neonatal asphyxia^b^	12 (40.0)	19 (19.4)	5.317	0.021^*^
	NRDS^b^	17 (56.7)	57 (58.2)	0.021	0.885
	BPD^b^	10 (33.3)	10 (10.2)	7.648	0.002^**^
	IVH^b^	12 (40.0)	18 (18.4)	5.990	0.014^*^
	Hydrocephalus^b^	1 (3.3)	0 (0)		0.234
	Brain damage in white matter^b^	3 (10.0)	1 (1.0)		0.040^*^
	Electroencephalogram abnormalities^b^	13 (43.3)	35 (35.7)	0.569	0.451
	Septicaemia^b^	10 (33.3)	13 (13.3)	6.276	0.012^*^
	Meningitis^b^	2 (6.7)	3 (3.1)		0.334
	NEC^b^	4 (13.3)	6 (6.1)	0.569	0.369
	ROP^b^	5 (16.7)	6 (6.1)	6.276	0.012^*^
Treatment during hospitalization	Hormones^b^	3 (10.0)	7 (7.1)	0.015	0.610
	Blood transfusion^b^	10 (33.3)	16 (16.3)	4.104	0.043^*^
	PS^b^	16 (53.3)	28 (28.6)	6.243	0.012^*^
	Levothyroxine^b^	14 (46.7)	21 (21.4)	7.545	0.006^**^
	Ventilator use^b^	23 (76.7)	71 (72.4)	0.209	0.647
	Invasive ventilator^b^	16 (53.5)	19 (19.4)	13.322	0.000^***^
	Surgery^b^	0(0.00)	6 (6.1)		0.335
	Length of stay in hospital (days)^a^	38.36 ± 24.49	29.24 ± 19.33	-1.869	0.069

Note: ^a^ is represented by $\overline x$*±s*, and the statistical value is the t value. B is expressed as the number of cases (%), and the statistical value is the $\times$2 value. P < 0.05 was considered statistically significant. * means P < 0.05, ** means P < 0.01, *** means P = 0.000. ARFs, amblyopia risk factors; GDM, gestational diabetes mellitus; SGA, small for gestational age; LGA, large for gestational age; NRDS, hyaline membrane disease of prematurity; PS, pulmonary surfactant; BPD, bronchopulmonary dysplasia; IVH, intraventricular hemorrhage; NEC, necrotizing enterocolitis of the newborn; ROP, retinopathy of prematurity

### Multifactorial analysis of risk factors in the neonatal period in children born prematurely for the corrected risk of amblyopia at 12 months of gestational age

On the basis of the risk factors found in Table 6 in the neonatal period, multifactorial logistic regression analysis was performed and the results revealed that the presence of BPD (odds ratio [OR] 8.355, 95% confidence interval [CI] 1.492, 46.786), brain white matter injury in preterm infants (OR 16.742, 95% CI 0.684, 409.804), treatment with levothyroxine (OR 2.859, 95% CI 0.946, 8.639), and use of an invasive ventilator (OR 2.983, 95% CI 0.942, 9.445), were independent risk factors for the corrected risk of amblyopia at 12 months of gestational age; hormone therapy (OR 0.055, 95% CI 0.004, 0.737) was found to be protective factors.

**Table 6:** Multifactorial logistic regression analysis of risk factors for amblyopia

Risk factor	Regression coefficient	Standard error	Wals value	P	OR	95% CI
Hormone regimen	-2.893	1.320	4.803	0.028	0.055	0.004–0.737
Invasive breathing (medicine)	1.093	0.588	3.453	0.063	2.983	0.942–9.445
BPD	2.123	0.879	5.833	0.016	8.355	1.492–46.786
White matter damage	2.818	1.632	2.983	0.084	16.742	0.684–409.804
Levothyroxine	1.051	0.564	3.468	0.063	2.859	0.946–8.639
Constant	-1.686	0.340	24.561	0.000	0.185	

BPD, bronchopulmonary dysplasia; OR, odds ratio; CI, confidence interval.

### Relationship between the amblyopia risk and neurological development in preterm infants with corrected gestational age of 12 months

Developmental abnormalities were present in nine (30%) children in the ARF group and 13 (13.27%) in the non-ARF group. The proportion of children with fine motor abnormalities was significantly higher in the ARF group than in the non-ARF group, with a significant difference (P = 0.034 < 0.05). There were no differences in the rates of adaptive, gross motor, language, and personal social abnormalities between the two groups of children. See Table 7 for details.

**Table 7:** Relationship between the risk factors for amblyopia and neurological development

Gesell Developmental Scales	ARF group (%)	Non-ARF group (%)	Chi-square (math.)	P
Adaptive	8 (26.7)	16 (16.3)	1.612	0.204
Large scale movement	12 (40.0)	22 (22.4)	3.627	0.057
Fine-motion	9 (30.0)	13 (13.3)	4.519	0.034*
Language	14 (46.7)	32 (32.7)	1.959	0.162
Personal social	8 (26.7)	15 (15.3)	2.011	0.156

Note: *P < 0.05 denotes statistical significant. ARFs, amblyopia risk factors

## Discussion

Premature infants are prone to childhood amblyopia. The results of this study show that among preterm infants who were hospitalized in the NICU and followed-up until 12 months of corrected gestational age, the prevalence of amblyopia risk was 24.0%, with astigmatism as the major risk factor for amblyopia. This study also showed that birth weight was significantly lower in the amblyopia risk group than in the non-amblyopia risk group. Analysis of factors during hospitalization in the neonatal period revealed that children in the amblyopia risk group had higher rates of PS use, blood transfusion, invasive ventilator use, BPD, IVH, cerebral white matter injury, sepsis, and levothyroxine use during hospitalization for pulmonary hyaline disease in preterm infants than in the non-amblyopia risk group, with significant differences (P < 0.05). Further multifactorial analysis revealed BPD, sepsis during hospitalization, and levothyroxine use as the independent risk factors for the risk of amblyopia at 12 months of gestational age, whereas glucocorticoid therapy for BPD was a protective factor for amblyopia.

Amblyopia is one of the leading causes of visual impairment in preschool children, and untreated amblyopia can have an impact on the academic life of the affected children [14]. People with amblyopia were less likely to complete higher education and had a three times higher risk of developing less than 6/12 vision in their better seeing eye in adulthood compared with people without amblyopia. Treatment of amblyopia also has the benefit of reducing the risk of future visual loss in both eyes due to the vision loss in the better seeing eye [15]. Screening for amblyopia early in life is associated with better healing of the child’s future vision. A 2008 study showed [16] that photoscreening for ARFs before the age of 2 years significantly reduced the proportion of children with visual acuity less than 20/40 after the age of 6 years. Thus, early screening is important for children at high risk of amblyopia.

The Spot photoscreener has high sensitivity as well as specificity for screening for amblyopia risk factors. According to previous studies, it has a sensitivity of 60–92.6% and specificity of 70.4–93% [17]. In children aged 12–23 months, it has also demonstrated a good sensitivity of 82.4% (56.6–96.2), specificity of 68.8% (50.0–83.9) [18], positive predictive value of 58.3 (36.6–77.9), and negative predictive value of 88.0 (68.8–97.5). This study used the Spot photoscreener for early screening of ARFs as it is suitable for use in the post-discharge follow-up of the delivered child with reliable results.

According to the results of this study, the percentage of children at risk of amblyopia was 23.4%. Previous studies have reported [19] that the proportion of risk factors for amblyopia in the normal population is 21 ± 2%, with astigmatism as the main risk factor, according to the American Association for Pediatric Ophthalmology and Strabismus (AAPOS) 2003 guidelines. The results obtained in the present study were consistent with the abovementioned findings. However, the AAPOS 2013 guidelines were used for the definition of ARFs in this study and the criteria were higher than those in the AAPOS 2003 guidelines (Table 8). This suggests that the percentages of ARFs obtained in the present study were higher than those in previous studies. Previous studies have shown a higher prevalence of amblyopia in preterm infants than in full-term children [20]. Gestational age < 27 weeks [21] was also a risk factor for amblyopia as was previous NICU hospitalization. The results of this study were different from those of previous studies, probably because our hospital is a transfer center for critically ill mothers and newborns in Beijing; therefore, preterm infants hospitalized in the NICU are relatively young for their gestational age, there are more low, very low, and ultra-low birth weight infants, and risk factors for amblyopia in this population are relatively high. Of the various risk factors, astigmatism was the most common risk factor, similar to the findings of previous studies.

**Table 8:** Comparison of the AAPOS 2003 guidelines with the AAPOS 2013 guidelines

	AAPOS 2003	AAPOS 2013
Anisometropia	> 1.5 D	> 2.50 D
Hyperopia	≥ 3.5 D	> 4.50 D
Astigmatism, not oblique	≥ 1.5 D	> 2.00 D
Astigmatism, oblique	≥ 1.0 D	> 2.00 D
Myopia	≥ 3.0 D	> 3.00 D
Strabismus	Manifest	> 8 PD

AAPOS, Association for Pediatric Ophthalmology and Strabismus

According to the results of this study, BPD, brain white matter damage in preterm infants, use of invasive ventilators, and levothyroxine were the independent risk factors for the corrected risk of amblyopia at the gestational age of 12 months. A study by Rudanko et al. [22] showed that children with very low birth weight (< 1500 g), gestational age (< 30 weeks), intrauterine infection, hyperbilirubinemia, respiratory disease, asphyxia, and prolonged use of ventilation faced increased risk of visual impairment. Similarly, a study by Hellström et al. [23] showed that extremely preterm birth, ROP, and severe BPD were important risk factors for ocular abnormalities in extremely preterm infants. However, previous studies have not grouped visual impairment in detail. Visual impairment in preterm infants may be neurodevelopment-related due to retinopathy, such as ROP or amblyopia. The results of the present study reveal the risk factors for determining the precise risk of amblyopia in children born prematurely.

This study indicates for the first time that BPD, thyroid replacement therapy for temporary hypothyroidism in preterm infants, and neonatal sepsis are independent risk factors for the risk of amblyopia. Among them, it was noted that the longer the use of invasive ventilators, the higher the incidence of BPD. The association between BPD and the use of invasive ventilators is consistent the findings of prior studies, and the occurrence of BPD represents the prolonged use of invasive ventilators and prolonged oxygen inhalation. Thus, clinicians should reduce the use of invasive ventilators and lower the concentration of inhaled oxygen as much as possible to help reduce the incidence of amblyopia in children.

Transient hypothyroidism in preterm infants is not uncommon clinically, especially in very low and ultra-low birth weight infants. This may be related to preterm infant nutrition during pregnancy as well as to disease and nutritional status after birth [24]. In contrast, during sepsis in the neonatal period, pathogens and inflammatory reactions caused by pathogens may cause damage to various organs, while critical conditions in children who are often exposed to other adverse factors such as high ambient lighting, phototherapy, and parenteral nutrition, may indirectly contribute to the increased risk of amblyopia. Meticulous clinical monitoring of infection indicators in the neonatal period to reduce sepsis, monitoring of thyroid function in preterm infants, and timely administration of levothyroxine replacement therapy are required.

In preterm infants with BPD, treatment with hormones can reduce the inflammatory lung response and decrease the severity of BPD. The results of this study show that hormonal treatment of preterm infants with BPD is a protective factor for the risk of amblyopia in children. It suggests that timely assessment of the occurrence of BPD and administration of hormonal therapy are beneficial not only for improving the pulmonary function of the child but also for the development of the child’s visual acuity. It is also worth noting that some studies have also suggested that systemic dexamethasone treatment in the early postnatal period (≤ 7 days after birth) may increases the rate of combined cerebral palsy or mortality, whereas the use of hydrocortisone does not. There is little evidence that the use of systemic corticosteroids in the late postnatal period (≥ 7 days after birth) has an impact on the incidence of cerebral palsy in late childhood [25]. Therefore, while actively evaluating treatment for BPD, attention needs to be paid to the type and timing of hormone use.

After correcting for the correlation between the risk of amblyopia and neurodevelopment at 12 months of gestational age, we found that children at risk of amblyopia had a significantly poorer fine motor development, which is consistent with the results of previous studies. O’Connor et al. showed [26] that visual acuity abnormalities in very low birthweight infants were associated with poorer fine motor development, and lower visual acuity was associated with poorer fine motor ability. This is because good visual acuity as well as binocular vision are prerequisites for a good fine motor performance. Patients with poor visual acuity and binocular vision impairment have a poorer fine motor ability than healthy subjects [27]. Binocular visual function is worse in children with amblyopia than in healthy children [28]. The results of a study by Webber et al. [29] showed that fine motor skills are weaker in children with amblyopia than in healthy children. In the assessment of neurodevelopment, adaptability, gross motor ability, language, and personal socialization were not associated with the risk of amblyopia, consistent with the clinical findings. This indicates the need for prompt clinical screening for ocular disease in children with fine motor deficits identified during the neurodevelopmental assessment of preterm infants during follow-up.

There are certain shortcomings of this study. First, the sample size of our study was relatively small. The outbreak of coronavirus disease 2019 (COVID-19) in early 2020 resulted in some children not being able to complete follow-up. We will conduct further studies with larger sample sizes in the future Second, this study was a risk-related study of amblyopia, and since the children included were young and amblyopia was not diagnosed, further longitudinal follow-up of preterm infants is needed to monitor the children’s visual development.

## Data Availability

The data that support the findings of this study are available on reasonable request from the corresponding author [Liu Yang]. The raw data are not publicly available due to [state restrictions, them containing information that could compromise research participant privacy/consent].
